# Effect of Byproducts of Flue Gas Desulfurization on the Soluble Salts Composition and Chemical Properties of Sodic Soils

**DOI:** 10.1371/journal.pone.0071011

**Published:** 2013-08-01

**Authors:** Jinman Wang, Zhongke Bai, Peiling Yang

**Affiliations:** 1 College of Land Science and Technology, China University of Geosciences, Beijing, People’s Republic of China; 2 Key Laboratory of Land Consolidation and Rehabilitation, Ministry of Land and Resources, Beijing, People’s Republic of China; 3 College of Hydraulic and Civil Engineering, China Agricultural University, Beijing, People’s Republic of China; University of Kansas, United States of America

## Abstract

The byproducts of flue gas desulfurization (BFGD) are a useful external source of Ca^2+^ for the reclamation of sodic soils because they are comparatively cheap, generally available and have high gypsum content. The ion solution composition of sodic soils also plays an important role in the reclamation process. The effect of BFGD on the soluble salts composition and chemical properties of sodic soils were studied in a soil column experiment. The experiment consisted of four treatments using two different sodic soils (sodic soil I and sodic soil II) and two BFGD rates. After the application of BFGD and leaching, the soil soluble salts were transformed from sodic salts containing Na_2_CO_3_ and NaHCO_3_ to neutral salts containing NaCl and Na_2_SO_4_. The sodium adsorption ratio (SAR), pH and electrical conductivity (EC) decreased at all soil depths, and more significantly in the top soil depth. At a depth of 0–40 cm in both sodic soil I and sodic soil II, the SAR, EC and pH were less than 13, 4 dS m^−1^ and 8.5, respectively. The changes in the chemical properties of the sodic soils reflected the changes in the ion composition of soluble salts. Leaching played a key role in the reclamation process and the reclamation effect was positively associated with the amount of leaching. The soil salts did not accumulate in the top soil layer, but there was a slight increase in the middle and bottom soil depths. The results demonstrate that the reclamation of sodic soils using BFGD is promising.

## Introduction

Wet flue gas desulfurization (FGD) is the dominant technology used in the control of SO_2_ emissions from coal-fired power plants. The major byproduct of the process is CaSO_4_, or a mixture of CaSO_3_ and CaSO_4_ [1]. With the rapid development of the energy and power industries in China, the installed capacity of power plants with FGD devices, and therefore the amount of byproducts of flue gas desulfurization (BFGD), is expected to increase rapidly. By the end of 2010, the installed capacity of power plants in China with FGD devices was about 57.8 GW, and the annual production of BFGD was about 7.1 million tons. According to the National Development Program of China, the installed capacity of power plants with FGD devices will be 530 GW by 2020, with an annual production of 90 million tons of BFGD. As BFGD contain large amounts of moisture and ash, they can only be used as building gypsum after purification and dehydration, hence they are more expensive compared with natural gypsum produced in China. If the BFGD were to be directly disposed of rather than being treated and used, a vast area of land would be required. Such an approach would be a waste of valuable land resources and represent a potential threat of secondary pollution to the environment [2]–[4].

Significantly, there are large areas of sodic soils in China. Excessive exchangeable sodium (ES) has gradually been adsorbed in soil colloid, causing deterioration in the soil’s physical properties and inducing the formation of sodic soils [5]. Sodic soils contain an excess of exchangeable Na^+^ in soil colloids and have soluble carbonates in the form of NaHCO_3_ and Na_2_CO_3_. The pH value, SAR and exchangeable sodium percentage (ESP) are greater than 8.5, 13 and 15, respectively, and the electrical conductivity of the saturated paste (EC_sat_) is less than 4.0 dS m^−1^ [5]–[6]. Amendments for sodic soils are materials that directly or indirectly supply divalent cations (usually Ca^2+^) to replace ES [3], [7]. The dissolution-exchange reaction in soil colloids is represented as [5].
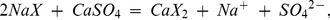
(1)where 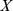
is the soil exchange phase. The exchange of Ca^2+^ for Na^+^ in soil colloid results in the flocculation of soil particles, which restores the porous structure and high permeability of the soil. Because the main component of BFGD is CaSO_4_, substantial evidence exists that these BFGD can, with proper use, be valuable for the reclamation of sodic soils [1], [3], [8]–[12].

The degree of ES absorbed by soil colloid depends on the soil soluble salts composition. ES is most easily absorbed into soil colloid when the soil solution contains large amounts of Na_2_CO_3_ and NaHCO_3_. In soil containing neutral salts (such as NaCl and Na_2_SO_4_), Na^+^ can only be absorbed by soil colloid and cause soil sodication if Na^+^/(Ca^2+^+Mg^2+^)≥4 in the cationic soil solution [13]. Therefore, the ion solution composition of sodic soils plays an important role in the reclamation process. Although several studies have examined the changes in the physicochemical properties of sodic soils and crop yield after applying BFGD, less attention has been paid to the ion solution composition [3], [6], [10], [12]–[13], [14]–[16].

The objective of this study was to determine whether the application of BFGD causes changes in the soluble salts composition, SAR, EC and pH value of sodic soils at different depths; to analyze the relationship between the soil’s chemical properties and soluble salts composition; and to elucidate the mechanism of sodic soil reclamation by BFGD.

## Materials and Methods

### Ethics Statements

This study was conducted with the permission of the Changsheng Experimental Station of the Baoyannur League Institute of Water Resources. No endangered or protected species were present in the field studies.

### The Physical and Chemical Properties of the Soils Tested and the BFGD

The soils tested were sampled from the Changsheng Experimental Station of the Baoyannur League Institute of Water Resources in northwestern China (N 40°20′, E 108°31′). Through on-site investigation, two different sodic soils (sodic soil I and sodic soil II) were sampled using spade. The soil sampling depth was divided into five layers (0–5 cm, 5–20 cm, 20–40 cm, 40–60 cm and 60–100 cm). The soils in different layers were air-dried, crushed, mixed and passed through a 2-mm sieve before the soil column experiments. The physical and chemical properties of the tested soils are listed in Table 1. The sodication degree of sodic soil II was higher than that of sodic soil I.

**Table 1:** Physical and chemical properties of the tested soils.

Soil type	Soil depth (cm)	Bulk density (g·cm^−3^)	pH	EC (dS·m^−1^)	ES (cmol·kg^−1^)	CEC (cmol·kg^−1^)	ESP (%)
Sodic soil I	0–5	1.41	8.95	11.45	1.65	17.14	9.63
	5–20	1.46	9.32	5.18	3.72	17.86	20.83
	20–40	1.41	9.29	4.31	2.44	16.95	14.38
	40–60	1.47	8.96	2.52	2.55	16.84	15.12
	60–100	1.44	8.96	2.31	2.19	16.71	13.12
Sodic soil II	0–5	1.58	9.07	13.09	9.46	21.23	44.55
	5–20	1.52	8.91	4.52	6.07	21.63	28.06
	20–40	1.46	9.23	1.89	5.60	20.15	29.74
	40–60	1.45	9.63	1.26	5.40	20.34	26.53
	60–100	1.46	9.44	0.84	4.91	19.86	24.71

The BFGD used in the test were from Huaneng Power International, Inc. The CaSO_4_ content in the by-product was 89.8% and the moisture content was almost 10.1% (Table 2). The concentrations of pollution elements were far below the tolerance limits regulated by the control standards for pollutants in fly ash for agricultural use (GB8173-87) and the control standards for pollutants in sludge for agricultural use (GB4284-84) [17]–[18].

**Table 2:** Component and properties of the BFGD.

pH	Density (g·cm^−3^)	Free water (%)	CaSO_4_·2H_2_O (%)		CaSO_4_·1/2H_2_O (%)		CaCO_3_ (%)
5.90	1.02	10.10	89.8		0.20		5.55
Cd (mg·kg^−1^)	Cr (mg·kg^−1^)	As (mg·kg^−1^)	Se (mg·kg^−1^)	Ni (mg·kg^−1^)	Cu (mg·kg^−1^)	Hg (mg·kg^−1^)	Pb (mg·kg^−1^)
0.01	83.4	5.04	4.24	21.35	83.26	0.32	99.38
5^a^	250^a^	75^a^	15^a^	200^a^	250^ a^	5.0^b^	250^a^

Note: ^a^Control Standards for Pollutants in Fly Ash for Agricultural Use (GB8173-87);

b Control Standards for Pollutants in Sludge for Agricultural Use (GB4284-84).

### The BFGD Application Rate

The principle underlying the reclamation of sodic soils by BFGD is that the calcium contained in the BFGD replaces the sodium in soil colloids. The application rate of the BFGD was determined using a modified method based on gypsum application rates [11], [15]. It was calculated according to the relative content of calcium in the BFGD and gypsum as follows:
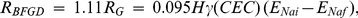
(2)where 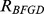 is the BFGD application rate in t hm^−2^; 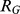 is the gypsum application rate in t hm^−2^; 1.11 is the modified coefficient, determined by comparing the content of CaSO_4_ in gypsum and BFGD; H is the soil depth to be reclaimed in cm, 20 cm in this study; 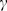 is the soil bulk density in g cm^−3^, and the values for sodic soil I and sodic soil II at a depth of 0–20 cm are shown in Table 1; 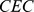 is the cation exchange capacity in cmol kg^−1^, and the capacities of sodic soil I and sodic soil II at 0–20 cm soil depth are shown in Table 1; 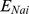
is the initial exchange sodium fraction, which is 1; and 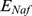
is the desired final exchange sodium fraction, which was 0.1 in this study.

### Experimental Design

The experiment consisted of four treatments (Table 3), with two sodic soils (sodic soil I and sodic soil II) and two BFGD rates. The BFGD application rates for sodic soil I and sodic soil II were divided into two levels. The amount of BFGD applied at the first level was determined according to the theoretical calculation of Formula 1. Taking into account the incomplete dissolution of the BFGD and deep percolation when it was applied, the amount of BFGD applied should be appropriately increased. The amount of the second application was determined by the theoretical calculation multiplied by a factor of 1.2. The BFGD application rates for different treatments are shown in Table 3 based on the initial properties of the sodic soil tested. The soil columns of four treatment were each leached four times.

**Table 3:** Experimental design.

Experimental treatment	BFGD application rate (g·kg^−1^)	BFGD application rate (t·hm^−2^) (g·kg^−1^)application rate (g·kg^−1^)	Sodic soils types
T1	3.00	1.65	Sodic soil I
T2	3.60	1.98	Sodic soil I
T3	7.00	3.86	Sodic soil II
T4	8.40	4.63	Sodic soil II

The columns tested were contained in unplasticized polyvinyl chloride (UPVC) plastic pipes, with an outside diameter of 16 cm, inside diameter of 15.2 cm and height of 100 cm. The bottom of the soil column was closed with a UPVC board with a 1-cm drainage hole. A 5-cm layer of quartz sand was placed in the bottom of each column as the filter layer. The columns were filled with treated soils according to the soil depth, and the volumetric soil moisture content was 10%. The weight of dry soil to be filled at different depths was determined according to soil bulk density. The soils were evenly mixed in a sealed plastic container with water that is 10% by volume of the soils to be filled and maintained two days, and the soils with 10% of volumetric water content were obtained. Soil bulk density and soil weight in different column layers are shown in Table 4. The soil was filled in 5-cm depth at each time and tamped to a desired height in respective layers. The surface soil was loosened before the next soil layer was filled. During the filling process, BFGD was applied to and mixed evenly with the soil at 0–5 cm depth and 5–20 cm soil depth in the amount specified in the experimental design (Table 3). The soil column leaching tests were then carried out.

**Table 4:** The bulk density and weight of soils filled in each soil depth.

Sodic soil I				Sodic soil II			
Soil depth (cm)	Bulk density (g·cm^−3^)	Soil Weight (kg)	Total weight (kg)	Soil depth(cm)	Bulk density(g·cm^−3^)	Soil Weight (kg)	Total weight (kg)
0–5	1.41	1.28	26.13	0–5	1.58	1.43	26.72
5–20	1.46	3.97		5–20	1.52	4.12	
20–40	1.41	5.13		20–40	1.46	5.28	
40–60	1.47	5.32		40–60	1.45	5.28	
60–100	1.44	10.43		60–100	1.46	10.61	

Because the soil water content in each soil column was only 10% (volumetric water content), the leaching water volume of each soil column was 4500 ml the first time (250 mm when translated into the field water volume; i.e. 2500 m^3^·hm^−2^), and was 1360 ml the second, third and fourth time (75 mm when translated into the field water volume; i.e. 750 m^3^·hm^−2^). The leaching interval was 12 d. The leaching soils were hierarchically sampled at soil depths of 0–5 cm, 5–10 cm, 10–20 cm, 20–40 cm, 40–60 cm, 60–80 cm and 80–100 cm. Each treatment soil column was sampled using an auger. The measured parameters were soluble salt composition (cations: CO_3_^2−^, HCO_3_^−^, Cl^−^ and SO_4_^2−^; anions: Ca^2+^, Mg^2+^, K^+^and Na^+^), pH, EC, ES and CEC.

### Analytical Methods and Statistical Analyses

The samples were air-dried and passed through a 1-mm sieve. The EC, pH, soluble anions and soluble cations were measured using 1∶5 water extracts. The soluble cations were measured using an atomic absorption spectrophotometer, soluble anions were determined by anion chromatography, and exchangeable cations were determined in 1 M ammonium acetate (pH = 7) extract. Following this extraction and washing with 96% alcohol, the cation exchange capacity was determined by the removal of ammonium ions by distillation [19]. Na and K were determined by flame emission spectroscopy in the extract, and Ca and Mg were determined by atomic absorption spectrophotometer. Soil pH was determined using the glass electrode method.

The SAR of 1∶5 soil to water extracts (SAR1∶5) was calculated as [5]

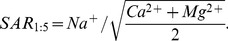
(3)


The physical and chemical items were measured twice, and the chemical analysis was replicated three times. The standard errors of the means of the three samples from each treatment were calculated. Descriptive statistics (mean, range, standard deviation (SD) and coefficient of variation (CV)) on soil parameters were analyzed with SAS9.1 (Statistical Analysis System; SAS Institute Inc.) and they were considered to be significantly different at *P*<0.05.

## Results

### Variations in the Soluble Ion Composition

**Figure 1:**
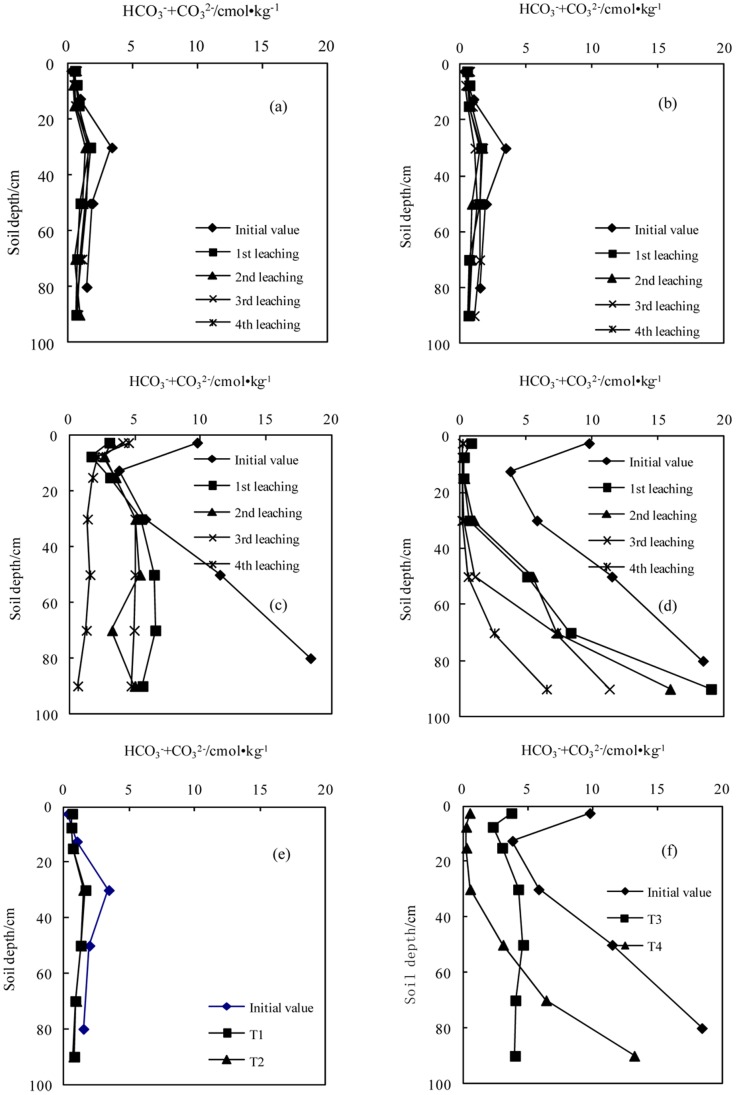
Variation in the concentration of carbonate and bicarbonate with successive leaching times (a.T1; b.T2; c.T3; d.T4); e. The average values in sodic soil I after leaching four times; f. The average value in sodic soil II after leaching four times).

**Figure 2:**
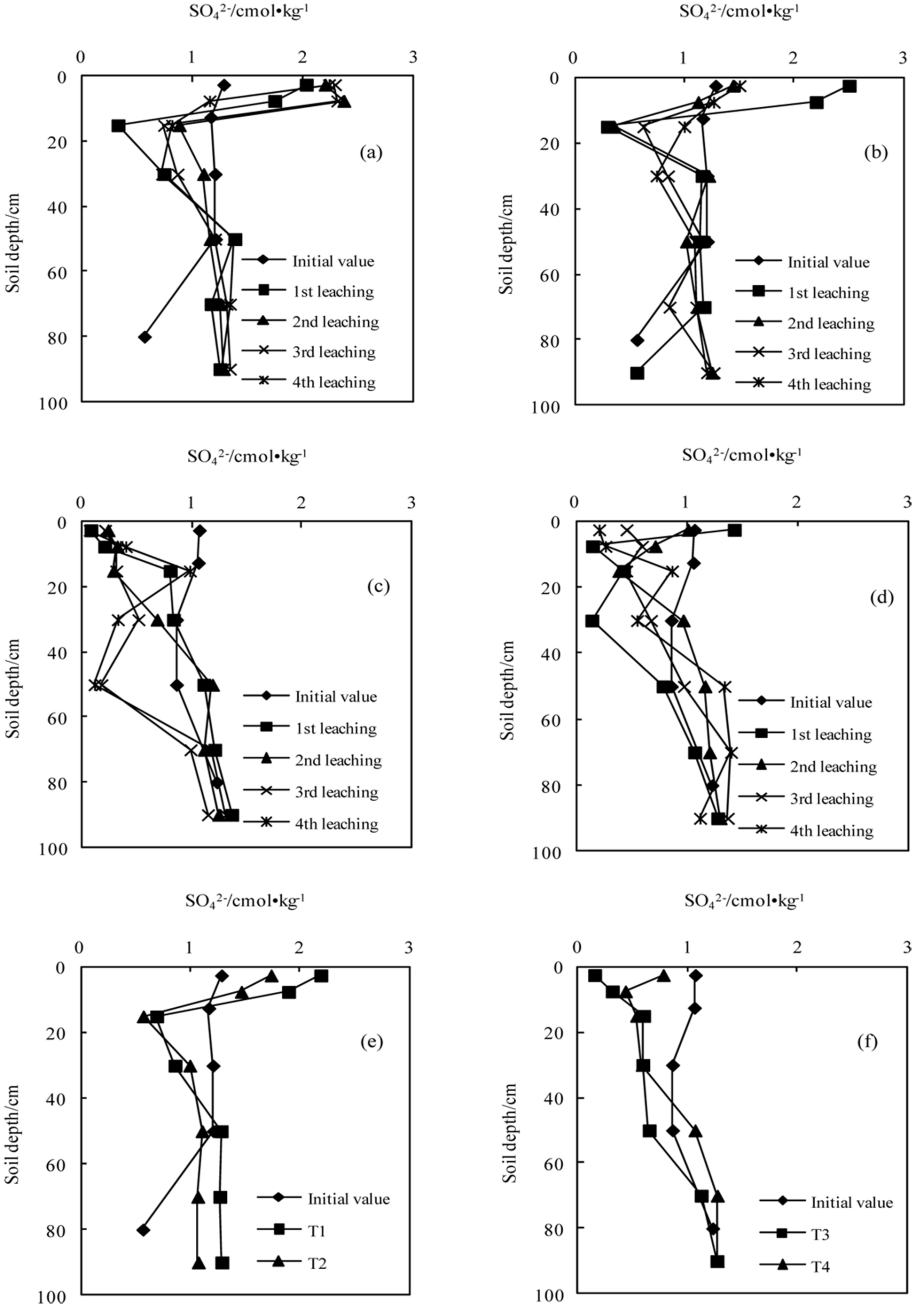
Variation in the concentration of sulfate with successive leaching times (a.T1; b.T2; c.T3; d.T4); e. The average values in sodic soil I after leaching four times; f. The average value in sodic soil II after leaching four times).

**Figure 3:**
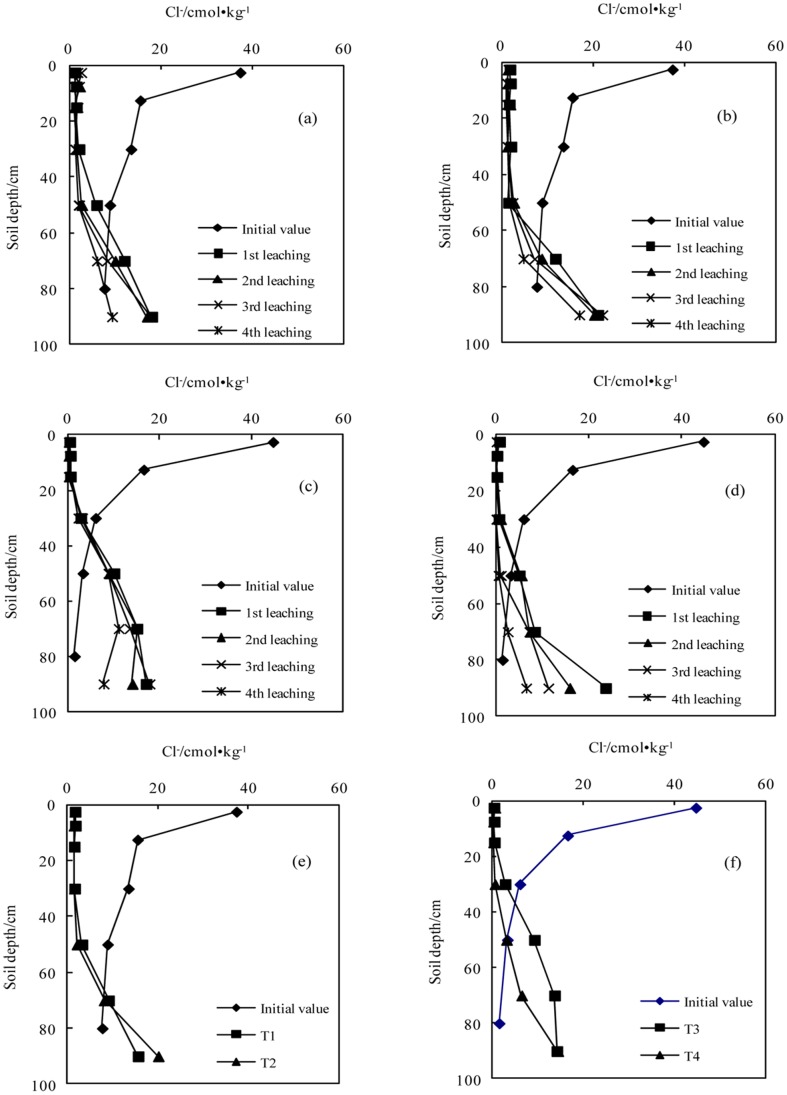
Variation in the concentration of chloride with successive leaching times (a.T1; b.T2; c.T3; d.T4); e. The average values in sodic soil I after leaching four times; f. The average value in sodic soil II after leaching four times).

**Figure 4:**
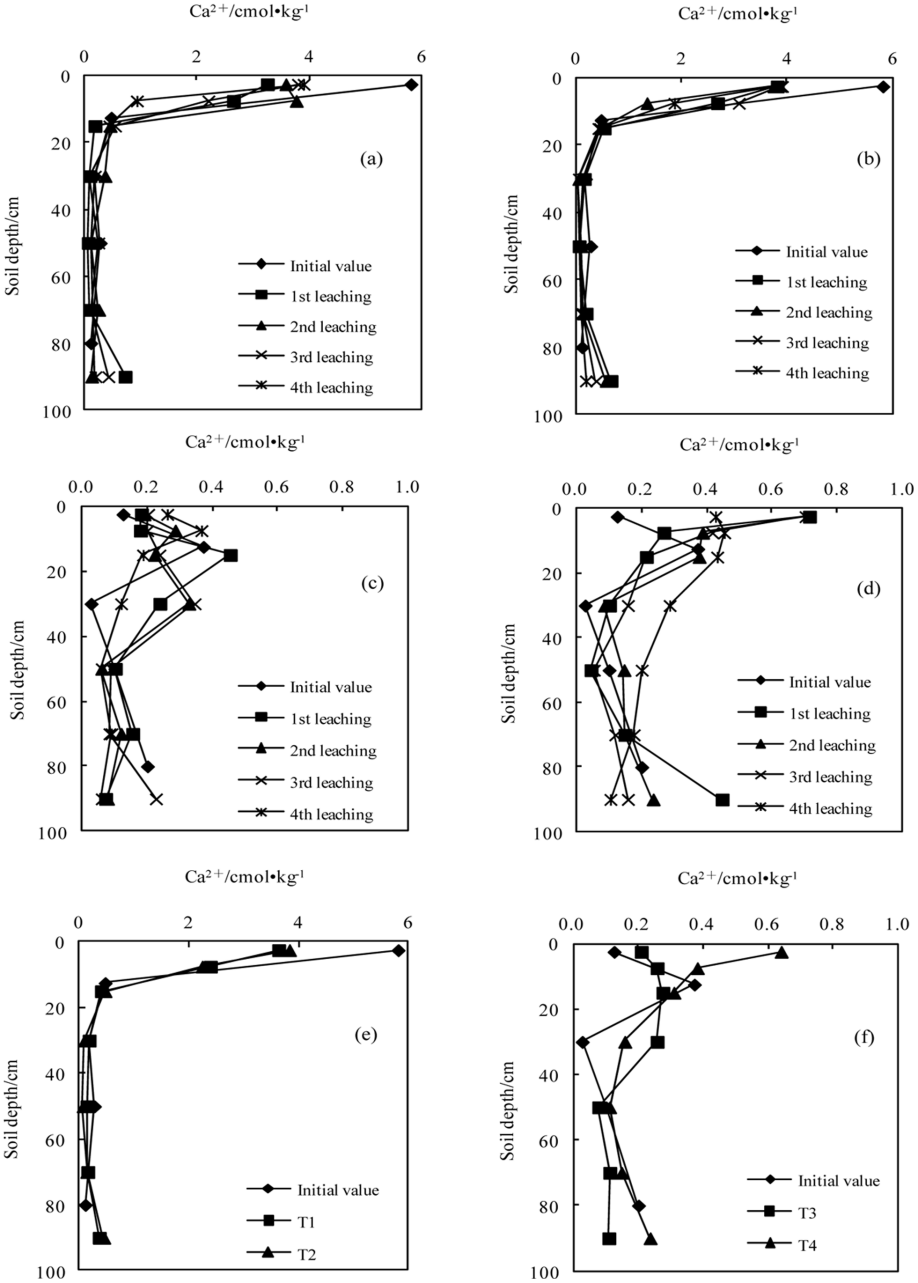
Variation in the concentration of calcium with successive leaching times (a.T1; b.T2; c.T3; d.T4); e. The average values in sodic soil I after leaching four times; f. The average value in sodic soil II after leaching four times).

**Figure 5:**
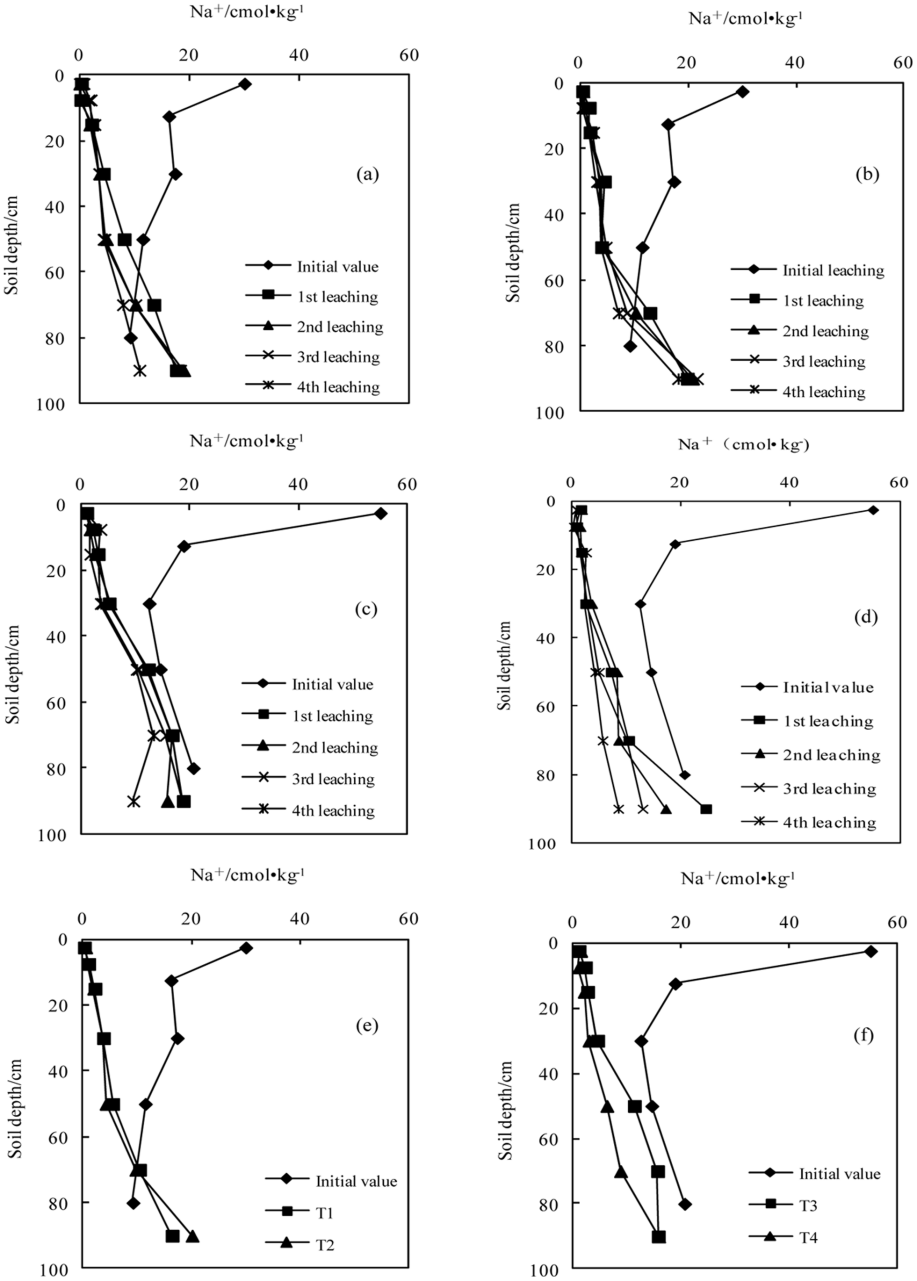
Variation in the concentration of sodium with successive leaching times (a.T1; b.T2; c.T3; d.T4); e. The average values in sodic soil I after leaching four times; f. The average value in sodic soil II after leaching four times).

After applying the BFGD to the sodic soils, the main component of BFGD, CaSO_4_, was constantly dissolved under the leaching action of the water flow, producing a rapid chemical reaction between the CaSO_4_ and the soluble salts in the soil solution. As the CaSO_4_ exchanged with the sodium in the soil colloid, it changed the soluble salts composition of the soil solution. The variation in the soluble salts composition (anions: HCO_3_^−^+CO_3_^2−^, SO_4_^2−^ and Cl^−^; cations: Ca^2+^ and Na^+^)of the soil solution are shown in Figs. 1–5.

#### The concentrations of HCO_3_^−^ and CO_3_^2−^

The concentrations of HCO_3_^−^ and CO_3_^2−^ in sodic soil II were higher than those in sodic soil I at the initial stage. After application of the BFGD, the concentrations of HCO_3_^−^ and CO_3_^2−^ in the two sodic soils decreased with each leaching, and the concentrations in the surface soils (0–30 cm) were lower than those in the deep soils (30–100 cm).

#### The concentrations of SO_4_^2−^

During the initial leaching stage, the concentration of SO_4_^2−^ at a depth of 0–20 cm in sodic soil I increased significantly, but there was no significant increase in sodic soil I. With each successive leaching, the concentration of SO_4_^2−^ gradually reduced, and that in sodic soil II at a depth of 0–20 cm also began to decrease. The concentrations of SO_4_^2−^ with a high BFGD application rate were higher than those with a low application rate at all soil depths in the two sodic soils.

#### The concentrations of Cl^−^

The concentration of Cl^−^ gradually reduced from the top to the bottom soils during the initial stage. After application of the BFGD and leaching, the Cl^−^ concentrations in the top soils of the two sodic soils decreased. The Cl^−^ accumulated in the bottom soil layer and gradually increased from the top to the bottom. The Cl^−^ concentrations with a high BFGD application rate were lower than those with a low application rate at all soil depths in the two sodic soils.

#### The concentrations of Ca^2+^

After application of the BFGD, the concentration of Ca^2+^ slightly increased in the top soil layer, but there was no obvious change in the other layers. The concentration of Ca^2+^ decreased significantly in the top soil layer with each subsequent leaching. The Ca^2+^ concentrations with a high BFGD application rate were higher than those with a low BFGD application rate at all soil depths in the two sodic soils.

#### The concentrations of Na^+^

The concentration of Na^+^ gradually reduced from the top to the bottom soils during the initial stage. After application of the BFGD and leaching, the concentration of Na^+^in each soil layer decreased, with a more obvious decrease in the top soil layer, with a gradual increase from the top to the bottom. The Na^+^ concentrations with a high BFGD application rate were lower than those with a low BFGD application rate at all soil depths in the two sodic soils.

### Variations in the Properties of the Sodic Soils

#### Soil SAR

**Figure 6:**
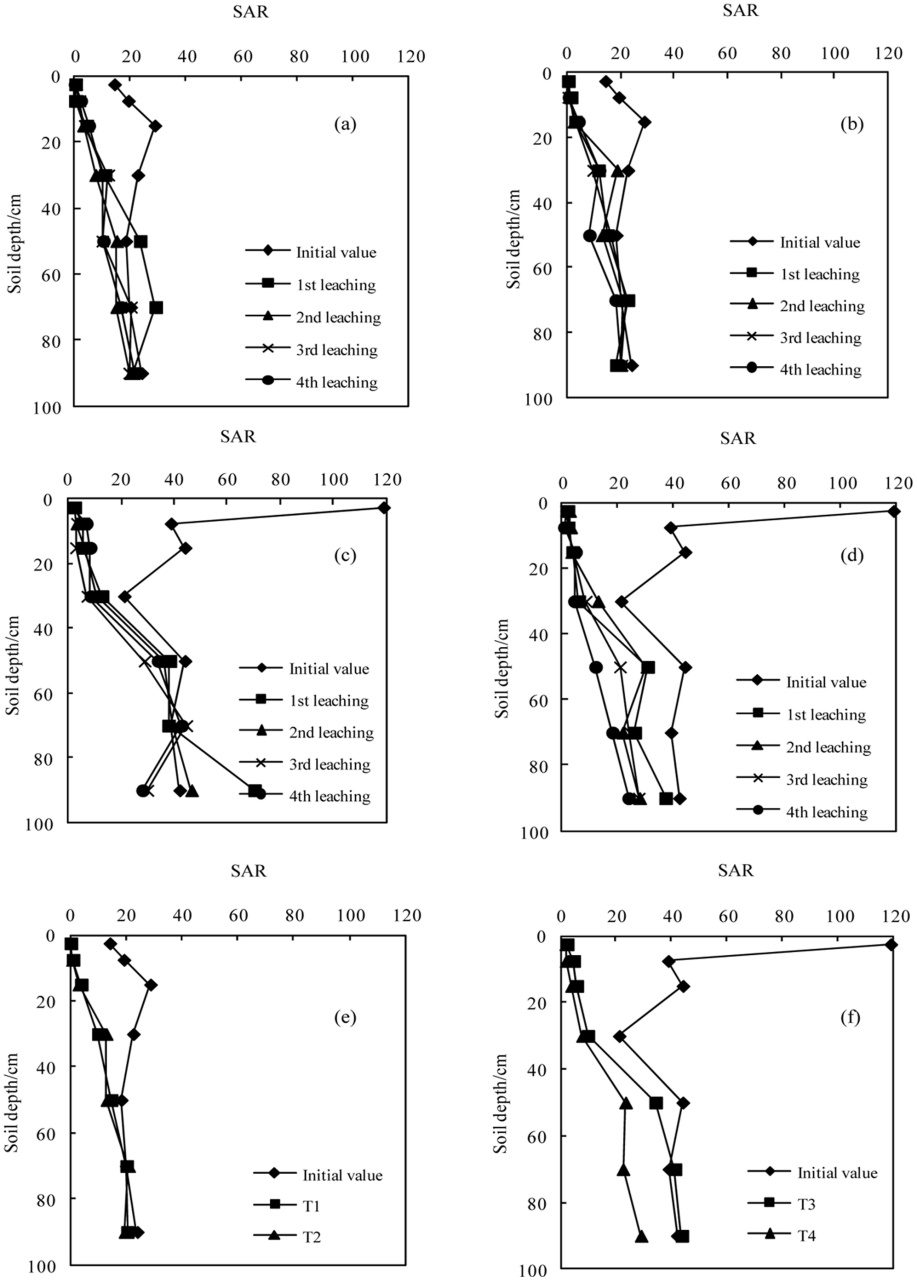
Variation in SAR with successive leaching times (a.T1; b.T2; c.T3; d.T4); e. The average values in sodic soil I after leaching four times; f. The average value in sodic soil II after leaching four times).

Under soil dispersion conditions, high SAR levels in the soil solution will cause severe soil clay dispersion and decrease hydraulic conductivity [20]. The variations in SAR following the application of BFGD are shown in Fig. 6. Leaching greatly reduced the SAR in the top soils, and to a much lesser extent in the bottom soils. The SAR was significantly reduced below the initial value at depths of 0–50 cm, 0–60 cm, 0–40 cm and 0–50 cm in T1, T2, T3 and T4 respectively, and the SAR was below 13 at depths of 0–50 cm, 0–50 cm, 0–30 cm and 0–30 cm respectively. For those soil depths at which the SAR was below the initial value, sodic soil I with a low ESP was greater than sodic soil II with a slightly higher ESP, and the treatments with high BFGD application rates were greater than those with low BFGD application rates. The extent of the SAR decrease was positively correlated with the amount of leaching. Under the same soil conditions, the reclamation effect improved with each leaching and the SAR showed an increasing trend from the top soil to the bottom soil. Under different treatments, the SAR of T2 was lower than that of T1 in sodic soil I and the SAR of T4 was lower than that of T3 in sodic soil II. The soil SAR was generally below the initial value in T4 at all depths, but only at a depth of 0–50 cm in T3. This indicates that the Na^+^was constantly being swapped and moved to the bottom layer of soil, thus increasing the relative proportion of Na^+^ in the bottom layer. Therefore, applying the BFGD and leaching reduced the Na^+^ in the soil solution by hindering the diffusion of the soil particles. Hence, the physical structure of the soil was improved and the sodic soil was reclaimed.

#### Soil EC

**Figure 7:**
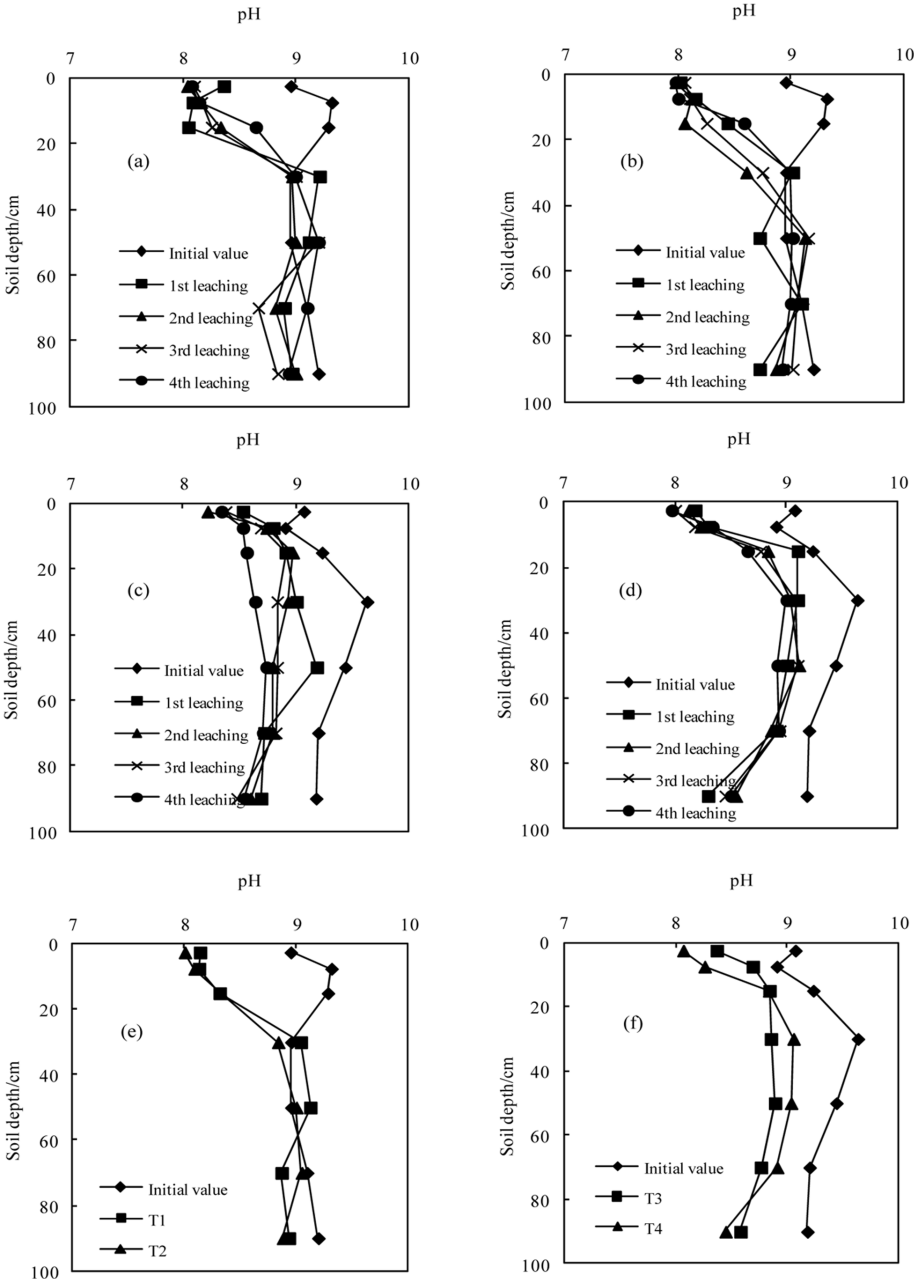
Variation in pH value with successive leaching times (a.T1; b.T2; c.T3; d.T4); e. The average values in sodic soil I after leaching four times; f. The average value in sodic soil II after leaching four times).

The variations in soil EC after the application of BFGD are shown in Fig. 7. The soil EC gradually increased from the top to the bottom soils during the initial stage. The EC in the surface soil significantly reduced under different treatments with the application of the BFGD and leaching, but increased in the bottom soil. The EC significantly decreased at depths of 0–60 cm, 0–60 cm, 0–40 cm and 0–40 cm in T1, T2, T3 and T4 respectively, and was below 4 dS m^−1^ at depths of 0–70 cm, 0–70 cm, 0–50 cm and 0–50 cm respectively. Therefore, application of the BFGD resulted in consistent improvement in the physical and chemical properties of sodic soils, and leaching did not cause an accumulation of salt in the surface soil. However, leaching should be strengthened to ensure the rapid discharge of salt accumulated in the bottom soil, prevent soil salinization and consolidate the reclamation effect on sodic soils.

#### Soil pH

**Figure 8:**
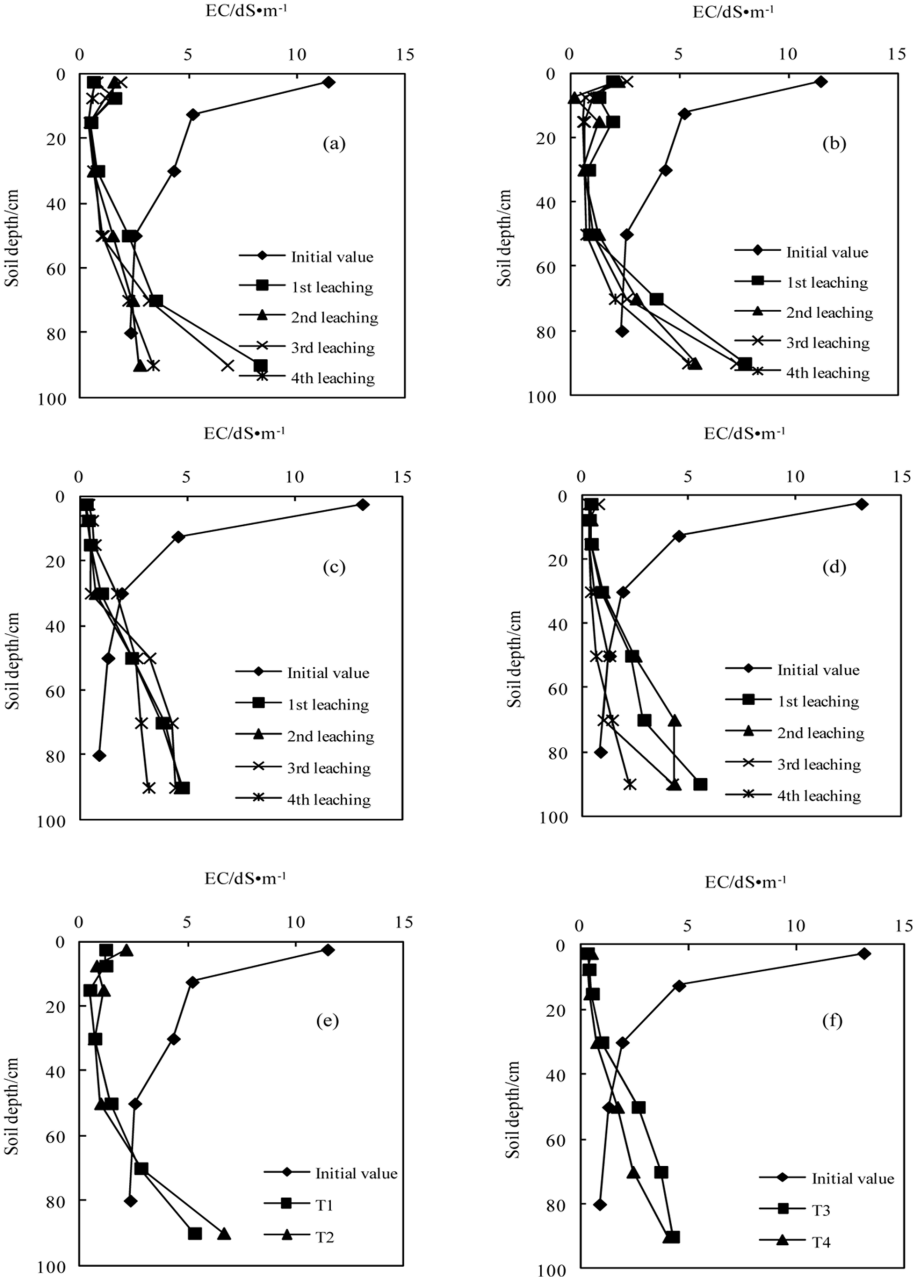
Variation in EC value with successive leaching times (a.T1; b.T2; c.T3; d.T4); e. The average values in sodic soil I after leaching four times; f. The average value in sodic soil II after leaching four times).

The variations in soil pH after the application of BFGD are shown in Fig. 8. The soil surface pH significantly reduced under different treatments with the application of the BFGD and leaching. The soil pH significantly decreased at depths of 0–40 cm, 0–40 cm, 0–20 cm and 0–20 cm in T1, T2, T3 and T4 respectively, and was below 8.5 at depths of 0–20 cm, 0–20 cm, 0–10 cm and 0–10 cm respectively. With different BFGD application rates, the pH value of T2 was less than that of T1 in sodic soil I at depths of 0–60 cm, but the value for T2 was higher than that for T1 at depths below 60 cm. The pH value of T4 was less than that of T3 in sodic soil II at depths of 0–20 cm, whereas the pH value of T4 was more than that of T3 in sodic soil II at depths of 20–100 cm. In the process of improving sodic soils, the Ca^2+^ concentration in the soil solution forms carbonate and precipitate due to excessive HCO_3_^−^ concentration in the irrigation water, thereby increasing the relative content of Na^+^in the soil solution [5], [16], [21]. The soil solution had a pH below 8.5, illustrating that the carbonate and bicarbonate had disappeared and the soluble salt had gradually made the transition from sodic salt to neutral salt, thus the sodic soils had been reclaimed.

## Discussion

### The Mechanism of Change in the Ionic Composition of Soluble Salts

The application of BFGD in sodic soils caused the main component of the BFGD, CaSO_4_, to dissolve due to the water flow infiltration. The CaSO_4_ comprised the dissolved part and the residual part, and the dissolved part reacted rapidly with the sodic salts (Na_2_CO_3_ and NaHCO_3_); see Equations 4 and 5 [5], [22]–[23].
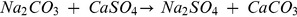
(4)

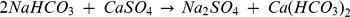
(5)


The concentrations of HCO_3_^−^ and CO_3_^2−^ rapidly decreased with these reactions, thus also reducing the soil pH value. After the disappearance of the sodic salt, there was an exchange reaction between the BFGD and the exchangeable Na^+^ in the soil colloids (see Equation 6), which generated the soluble salt sodium sulfate [3]. As the sodium sulfate leached into the deep soil layer, the sodic level of the soil continuously decreased.
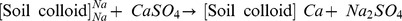
(6)


The application of the BFGD should increase the concentration of SO_4_^2−^ and Ca^2+^due to the dissolution of CaSO_4_. However, because the solubility of CaSO_4_ is relatively low, only 2 g·l^−1^ (25°C), the dissolved Ca^2+^ reacted with the sodic salts in the soil solution and the exchangeable Na^+^ in the soil colloids, thus consuming the Ca^2+^ in the soil solution. The chemical reaction between SO_4_^2−^ and Na^+^ generated soluble sodium sulfate, which was then leached into the deeper soil levels. Therefore, rather than increasing the concentrations of Ca^2+^and SO_4_^2−^, the concentrations were actually lower than the initial values in some soil layers. This effect is predominantly due to the increased permeability of the sodic soil once the salt has been leached from it and the concentration of Ca^2+^ and SO_4_^2−^ has decreased [3], [6], [16], [24]–[26].

In sodic soil areas there is also a certain amount of salinization, in which the soils contain some salt and the NaCl content is often very high. NaCl has a salting effect on the BFGD, which improves the solubility of CaSO_4_, increases the infiltration characteristics of sodic soils and accelerates their reclamation [5], [26]–[30]. With the reclamation of sodic soils, NaCl was constantly leached from the soil, thus reducing the NaCl content in the surface soil, and accumulating it in the deeper soil layers. There was a decrease in the Cl^−^ content at depths of 0–60 cm and 0–40 cm for sodic soil I and sodic soil II respectively, and an increase in the Cl^−^ content at depths of 60–100 cm and 40–100 cm.

As a result, the soil soluble salts made the transition from sodic salts containing Na_2_CO_3_ and NaHCO_3_ to neutral salts containing NaCl and Na_2_SO_4_. Leaching of the soluble salts from the soil resulted in continuous improvement in the sodic soils.

### The Relationship between Soil Properties and Soluble Salts Composition

The BFGD also immediately increased the EC of the soil solution and increased the ionic strength, which leads to proton generation and a reduction in the soil pH value [31]. The higher BFGD application rate reduced the soil SAR significantly more efficiently than the lower BFGD application rate because the BFGD increased Ca^2+^ in the soil solution, which promoted the displacement of the adsorbed Na^+^ and subsequent leaching. A decrease in SAR_e_ was also found in the control treatment. This was attributed to the “valence dilution” mechanism reported by Reeve and Bower [32]. In a soil-water system, there is equilibrium between the monovalent and divalent cations at the soil exchange site and in the solution. The equilibrium is altered by adding water to the system. This dilution of the soil solution favors the adsorption of divalent cations such as Ca^2+^ at the cost of monovalent cations such as Na^+^. Furthermore, if the soil solution is diluted by a factor of ΔC while maintaining the same ionic ratios, the initial SAR decreases by the factor (ΔC)^1/2^, i.e.,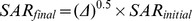. Because of the dilution, the (ΔC)^ 1/2^ is less than 1. Thus, the *SAR _final_* is less than the *SAR_initial_*.

The application of BFGD was also found to be effective in lowering the soil pH. The significant decrease in soil pH may be attributed to leaching of the exchangeable Na^+^, which was replaced by Ca^2+^ from the BFGD, and the decreased concentrations of HCO_3_^−^ and CO_3_^2−^. This pH decrease was partly associated with the degree of soil reclamation and especially with the reduction in soil sodicity [5], [33]. The pH of sodic soils is a function of the activity of CO_3_^2−^+HCO_3_^−^, and partially due to CO_2_ and ionic strength [34]. The presence of soluble Na+ increases the pH of calcareous soils by increasing the activity of carbonate and bicarbonate. Sodium carbonate (Na_2_CO_3_) and sodium bicarbonate (NaHCO_3_) are relatively soluble, and their removal by leaching can substantially reduce pH. Thus, the decrease in pH was in part associated with the leaching of soluble salts [35]–[36].

### Conclusions

The following conclusions can be drawn from our findings.

1. The application of BFGD and leaching caused the soil soluble salts to make the transition from sodic salts containing Na_2_CO_3_ and NaHCO_3_ to neutral salts containing NaCl and Na_2_SO_4_. The poor physical properties of sodic soils meant that long leaching times were needed to obtain good reclamation effects.
2. The soil SAR, pH and EC decreased at all soil depths, although it was more significant in the top soil. At a depth of 0–40 cm, the soil SAR, EC and pH were reduced to less than 13, 4 dS m^−1^ and 8.5 respectively, in both sodic soils I and II.
3. Leaching played a key role in the reclamation process, and the reclamation effect was positively correlated with the amount of leaching. The soil salts did not accumulate in the top soil layer, and there was a slight increase in the soil salts in the middle and bottom layers. Stronger leaching is thus required to ensure the salt accumulated in the bottom soil is discharged rapidly, thus preventing soil salinization and consolidating the reclamation effect.
4. The use of BFGD is inefficient, and the reclamation effect was improved by increasing the BFGD application rate by 20% above the theoretically calculated base rate.
5. The results of this research demonstrate that the reclamation of sodic soils using BFGD is promising.
